# A rare case of right proximal subclavian artery aneurysm: a case report

**DOI:** 10.1093/jscr/rjaf430

**Published:** 2025-06-18

**Authors:** Dianzhu Ding, Guangwei Jiang, Xiaoming Shi

**Affiliations:** Department of Vascular Surgery, Hebei General Hospital, No. 348 Heping West Road, Shijiazhuang 050051, Hebei, China; Department of Vascular Surgery, Hebei General Hospital, No. 348 Heping West Road, Shijiazhuang 050051, Hebei, China; Department of Vascular Surgery, Hebei General Hospital, No. 348 Heping West Road, Shijiazhuang 050051, Hebei, China

**Keywords:** aneurysm, subclavian artery, endovascular procedure, emergency procedure

## Abstract

Aneurysms in the subclavian artery are rare. In this article, chest pain and dyspnea were reported as the first symptoms of a giant right subclavian artery true aneurysm. A 75-year-old female patient was admitted because of intermittent chest pain and dyspnea for 2 weeks. Computed tomography revealed a large gourd-shaped right proximal subclavian artery aneurysm (SAA). She had the signs of right chest pain which is identified as pre-rupture of aneurysm. Additionally, the patient was experiencing obvious symptoms of airway obstruction. A variable-diameter-covered stent was implanted through the right common carotid artery and brachiocephalic artery by an emergency surgery. After 26 months’s follow-up, the patient was living well with no symptom of chest pain or dyspnea. A descending diameter covered stent implanted through right common carotid artery approach would be a proper method for the treatment of giant SAA.

## Introduction

Subclavian artery aneurysms (SAAs) are rare but potentially life-threatening due to the risk of rupture. In recent years, endovascular treatment using stent-grafts has emerged as a viable alternative to open repair. However, an adequate proximal and distal neck of the aneurysm, which serves as landing zones for the stent-graft, is essential. This report presents a case of a giant SAA that lacked a proximal neck, which was successfully treated with endovascular repair via the right common carotid artery and the right brachial artery.

## Case report

**Figure 1:**
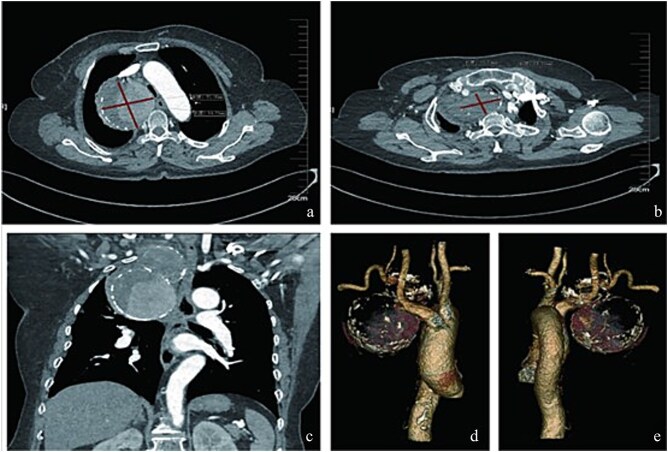
Radiologic characteristics of the SAA. (a) Axial CTA demonstrates a bipartite aneurysm morphology with superior–inferior compartmentalization. The inferior spherical component measures 70.05 mm (long axis) × 69.73 mm (short axis), exhibiting regular contour and homogeneous attenuation. (b) The superior ellipsoid component displays dimensions of 51.81 mm (long axis) × 35.59 mm (short axis), with peripheral thrombus formation. (c) Coronal reformation reveals the characteristic gourd-shaped configuration and intraluminal thrombus burden. (d and e) Volume-rendered reconstructions delineate circumferential calcifications along the aneurysm wall, particularly prominent at the inflow/outflow transitional zone.

A 75-year-old female patient was admitted because of intermittent chest pain and dyspnea for 2 weeks. A thoracic aorta computed tomography angiography (CTA) demonstrated a large right proximal SAA with partial thrombosis, and the adjacent trachea, esophagus, and blood vessels were obviously compressed and displaced (Fig. 1). Physical examination revealed wheezing and phlegm sounds in the chest, and no obvious pulsatile mass was found in the right supraclavicular fossa.

**Figure 2:**
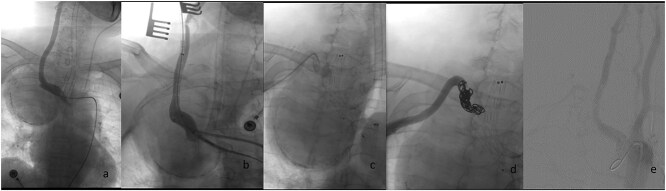
Endovascular procedural steps. (a) Pre-interventional angiography identifies the SAA originating from the ostium of the right subclavian artery. (b) Fluoroscopic guidance demonstrates retrograde deployment of the tapered stent-graft through the common carotid artery approach, with real-time angiography confirming appropriate stent positioning. (c) Post-stent angiography reveals residual aneurysm cavity perfusion through persistent outflow tracts. (d) Selective embolization of the outflow artery using detachable coils (chevrons) achieves complete cavity obliteration. (e) Final control angiography documents technical success: Preserved patency of the brachiocephalic trunk and right common carotid artery, absence of endoleak, and delayed opacification of the distal subclavian artery via collateral circulation.

Considering the serious symptoms of chest pain and dyspnea, which indicated that the SAA might rupture at any time and could cause acute airway obstruction, emergency surgery was performed. The procedure was as follows (Fig. 2): Angiography was conducted immediately following a retrograde right femoral artery approach. The angiography showed a gourd-shaped calcification near the mediastinum in the upper lobe of the right lung, and the brachiocephalic trunk artery (BA) and the right common carotid artery (CCA) were well visualized. An SAA erupted from the right beginning of the right subclavian artery, and the aneurysm was gourd-shaped; the inflow artery was at the lower layer, and the outflow artery was at the upper layer. Based on these findings, we decided to place a covered stent through the BA and CCA. However, the diameter of the BA was 13 mm and the CCA was 8 mm. The normal covered stents were of equal diameter. Therefore, we decided to use a descending diameter covered stent, Endurant II LIMBS, which is commonly used in the endovascular treatment of abdominal aortic aneurysms. As the delivery length of the stent is only 57 cm, we could not place the stent through the femoral artery. The CCA was the suitable operative approach. The right CCA was dissected out, a retrograde puncture of the right CCA was performed, and a 14F introducer sheath was placed. A 16 mm–10 mm–93 mm Endurant II LIMBS (Medtronic Ireland, Parkmore Business Park West, Galway, Ireland) was implanted through the CCA approach. Immediately after the deployment of the stent grafts, angiography was performed, and no endoleak was observed. The right common carotid artery was sutured with a 6–0 Prolene suture. To assess the condition of the upper aneurysm, angiography was performed through the right brachial artery. The partial cavity of the aneurysm could be visualized, but the origins of the vertebral artery and internal thoracic artery were close to the aneurysm. Therefore, coil embolization of the outflow artery was performed. Considering that this side of the vertebral artery was not dominant, the origin of the right vertebral artery was not protected. Angiography was performed after embolization; no endoleak was found, the BA and the right CCA were well visualized, and the distal subclavian artery was visualized later through the collateral circulation.

**Figure 3:**
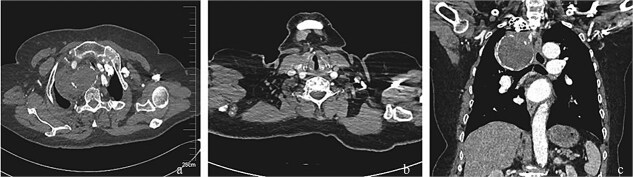
Post-interventional imaging surveillance at 4-month follow-up. (a) Axial CTA demonstrates complete thrombosis of the aneurysm sac following successful flow isolation, with volume reduction compared to pretreatment dimensions. (b) Sagittal reconstruction confirms absence of right vertebral artery opacification, consistent with deliberate ostial exclusion during the index procedure. (c) Coronal volume-rendered technique delineates organized thrombus within the collapsed aneurysm cavity, patency of the stent-graft lumen, and preserved distal subclavian artery perfusion through collateral channels. The radiopaque coil mass at the outflow tract confirms successful embolization endpoint achievement.

Postoperative management included daily aspirin (100 mg). The patient demonstrated no clinical evidence of distal limb ischemia or cerebrovascular events during the early postoperative period. At the 4-month postoperative follow-up, a surveillance thoracic aorta CTA performed at our institution confirmed complete aneurysm exclusion with satisfactory stent-graft apposition and absence of endoleak (Fig. 3). Longitudinal monitoring extended over 26 months revealed durable therapeutic efficacy. Importantly, the most recent telephone-based follow-up evaluation demonstrated persistent symptom remission, with no reported recurrence of chest pain, dyspneic episodes, or neurological deficits indicative of thromboembolic complications.

## Discussion

Etiologies of subclavian aneurysms include thoracic outlet syndrome, trauma, atherosclerosis, fibromuscular dysplasia, arterial inflammatory disease, and infection [1]. Clinical manifestations include chest pain, dyspnea, hoarseness, a pulsating mass in the neck, and vertebrobasilar ischemic events. It is easy to misdiagnose these conditions as a lung tumor in X-ray or CT examinations, so Doppler ultrasonography, chest-enhanced CT, or arterial CTA must be combined to make a definite diagnosis. For subclavian aneurysms, open surgery includes simple subclavian artery ligation, subclavian aneurysm resection, and artificial vascular reconstruction. Open surgery is still associated with a high rate of mortality and complications as a classic treatment [2]. Endovascular interventional therapy and hybrid surgery are becoming the main treatments for subclavian aneurysms in recent years due to their low complication rate and good short-term and long-term efficacy [3].

This case illustrates two critical considerations in SAA management. First, the significant BA-CCA diameter gradient (13 mm vs. 8 mm) precluded conventional cylindrical endografts, necessitating the use of a tapered Endurant II LIMBS stent. This adaptation achieved secure proximal and distal sealing while mitigating stent infolding risks in the narrower CCA. Second, the observed dyspnea resolution likely resulted from synergistic mechanisms: mechanical decompression through post-procedural hematoma resorption reduced tracheal compression, while hemodynamic stabilization via laminar flow restoration diminished pulsatile irritation of adjacent neural structures such as the recurrent laryngeal nerve.

The 26-month asymptomatic follow-up period supports the durability of this approach, but long-term monitoring remains imperative. This experience highlights that tapered endograft deployment via CCA access represents a viable option for giant right SAAs with hostile anatomy. The retrograde CCA approach was selected for this case based on the following considerations: First, during the transfemoral approach, the use of extra-stiff guidewires is often required to enhance support for endograft stability during long-path delivery. However, the inherent rigidity of these guidewires may elevate the risks of guidewire tip migration and dislodgement of unstable plaques, potentially increasing perioperative stroke risk. Second, in this elderly female patient, aortic arch morphology combined with vascular degenerative changes significantly compromises the deliverability of the antegrade system. Furthermore, compared to the long-distance antegrade approach, the retrograde CCA access provides superior precision in endograft deployment. Successful implementation requires meticulous preoperative planning with CTA to quantify vascular diameter discrepancies, verification of vertebral artery dominance patterns, and hybrid operative capabilities for access conversion. While this strategy effectively balances procedural safety with anatomic constraints, further multicenter studies are warranted to standardize device selection criteria and validate long-term outcomes.
